# Contribution of Altered Endocannabinoid System to Overactive mTORC1 Signaling in Focal Cortical Dysplasia

**DOI:** 10.3389/fphar.2018.01508

**Published:** 2019-01-09

**Authors:** Daniel García-Rincón, Javier Díaz-Alonso, Juan Paraíso-Luna, Zaira Ortega, José Aguareles, Adán de Salas-Quiroga, Cristina Jou, Inmaculada de Prada, Verónica Martínez-Cerdeño, Eleonora Aronica, Manuel Guzmán, María Ángeles Pérez-Jiménez, Ismael Galve-Roperh

**Affiliations:** ^1^Instituto Ramón y Cajal de Investigación Sanitaria, Department of Biochemistry and Molecular Biology and Instituto Universitario de Investigación Neuroquímica, Complutense University, Madrid, Spain; ^2^Centro de Investigación Biomédica en Red sobre Enfermedades Neurodegenerativas, Madrid, Spain; ^3^Departamento de Anatomía Patológica, Hospital Sant Joan de Déu, Barcelona, Spain; ^4^Hospital Infantil Universitario Niño Jesús, Madrid, Spain; ^5^Institute for Pediatric Regenerative Medicine, Shriners Hospital for Children of Northern California and Department of Pathology and Laboratory Medicine, School of Medicine, University of California, Davis, Sacramento, CA, United States; ^6^Amsterdam UMC, Department of (Neuro)Pathology, Amsterdam Neuroscience, University of Amsterdam, Amsterdam, Netherlands; ^7^Stichting Epilepsie Instellingen Nederland, Heemstede, Netherlands

**Keywords:** cannabinoid, CB_1_ receptor, malformation of cortical development, corticogenesis, neural progenitor, cannabinoid, mTORC1, mammalian target of rapamycin

## Abstract

Alterations of the PI3K/Akt/mammalian target of rapamycin complex 1 (mTORC1) signaling pathway are causally involved in a subset of malformations of cortical development (MCDs) ranging from focal cortical dysplasia (FCD) to hemimegalencephaly and megalencephaly. These MCDs represent a frequent cause of refractory pediatric epilepsy. The endocannabinoid system -especially cannabinoid CB_1_ receptor- exerts a neurodevelopmental regulatory role at least in part via activation of mTORC1 signaling. Therefore, we sought to characterize the possible contribution of endocannabinoid system signaling to FCD. Confocal microscopy characterization of the CB_1_ receptor expression and mTORC1 activation was conducted in FCD Type II resection samples. FCD samples were subjected to single nucleotide polymorphism screening for endocannabinoid system elements, as well as CB_1_ receptor gene sequencing. Cannabinoid CB_1_ receptor levels were increased in FCD with overactive mTORC1 signaling. CB_1_ receptors were enriched in phospho-S6-positive cells including balloon cells (BCs) that co-express aberrant markers of undifferentiated cells and dysplastic neurons. Pharmacological regulation of CB_1_ receptors and the mTORC1 pathway was performed in fresh FCD-derived organotypic cultures. HU-210-evoked activation of CB_1_ receptors was unable to further activate mTORC1 signaling, whereas CB_1_ receptor blockade with rimonabant attenuated mTORC1 overactivation. Alterations of the endocannabinoid system may thus contribute to FCD pathological features, and blockade of cannabinoid signaling might be a new therapeutic intervention in FCD.

## Introduction

Malformations of cortical development are associated with refractory epilepsy in children and young adults (Iffland and Crino, 2017). These alterations are originated by the disruption of key processes during brain development, such as neural progenitor cell proliferation or neuronal migration. Within MCD, some types of FCD and TSC are associated with overactivation of the mTORC1 signaling pathway (Aronica and Crino, 2014; Najm et al., 2018). TSC has a clear genetic origin on loss-of-function mutations of mTORC1 signaling regulators such as TSC1/TSC2 (hamartin–tuberin), which leads to the overactivation of mTORC1. Whereas the origin of the different FCD subtypes is still unclear, recent research has demonstrated the involvement of alterations of the PI3K/Akt/mTORC1 pathway particularly in FCD Type II (Jansen et al., 2015; Lim et al., 2015; D’Gama et al., 2017). Characteristic features of FCD Type II are the presence of balloon cells (BCs) and cytomegalic neurons with overactive mTORC1 signaling (usually revealed by the phosphorylation of one of its canonical targets, the ribosomal protein S6). Hence, FCD Type II cases are distinguishable at the molecular level from FCD Type I by the overactivation of mTORC1 signaling. FCD Type II cases are also distinguishable from Type I cases by the expression of undifferentiated cell markers in BCs that suggests a developmental origin (Orlova et al., 2010). Thus, this subtype of FCDs and hemimegalencephaly are known to be originated in the dorsal telencephalic progenitors and excitatory projection neuron lineage (D’Gama et al., 2017; Iffland et al., 2018). Further investigation on the mechanisms responsible for the epileptogenic network is required for the development of novel therapeutic strategies aimed to manage FCDs.

The ECS, and especially the cannabinoid CB_1_ receptor, exerts an essential neuromodulatory role in the adult brain via the retrograde lipid messengers 2-arachidonoylglycerol and anandamide (Soltesz et al., 2015). In addition, CB_1_ receptors are expressed during human cortical development (Mato et al., 2003; Wang et al., 2003). During early stages in brain development, endocannabinoids act as neural cell fate regulatory cues, and the CB_1_ receptor is coupled to activation of the mTORC1 pathway, which allows the control of neural progenitor identity and pyramidal neuron generation (Díaz-Alonso et al., 2015). Cannabinoid signaling regulates long-range axon projection (Mulder et al., 2008; Argaw et al., 2011; Díaz-Alonso et al., 2012), but also local microcircuits and interneuron development (Berghuis et al., 2005, 2007). Moreover, CB_1_ receptors are required for proper radial migration during cortical development, and their genetic inactivation induces brain hyperexcitability (Díaz-Alonso et al., 2017). Interestingly, transient loss of CB_1_ receptor function (induced by pharmacological down-regulation or small-interfering RNA) during embryonic development exerts long-lasting alterations in cortical development that result in increased seizure susceptibility in the adult offspring (de Salas-Quiroga et al., 2015; Díaz-Alonso et al., 2017). CB_1_ receptors are expressed in FCD (Zurolo et al., 2010), but their functional relevance is unclear. Thus, here we sought to investigate the potential contribution of aberrant cannabinoid signaling to FCD developmental pathogenesis.

## Materials and Methods

### Human Samples

The FCD cases included in this study were obtained from the archives of the Departments of Neuropathology of the Academic Medical Center (University of Amsterdam, Netherlands), the University Medical Center in Utrecht (Netherlands), Hospital Infantil Niño Jesús (Madrid, Spain) and Biobanc de l’Hospital Infantil Sant Joan de Déu per a la Investigació (Barcelona, Spain) integrated in the Spanish Biobank Network of ISCIII. A total of 30 surgical specimens (10 FCD Type I and 20 FCD Type II), resected from patients undergoing surgery for intractable epilepsy, were examined (Table 1). Tissue was obtained and used in accordance with the Declaration of Helsinki and informed consent was obtained for the use of brain tissue and for access to medical records for research purposes. All cases were reviewed by the corresponding neuropathologist and the diagnosis was confirmed according to ILAE classification system (Blümcke et al., 2011; Najm et al., 2018). Control brains from patients that were not diagnosed with neurologic disorders were also employed [*n = 6;* age (years): 53; 40; 34; 21; 51; 25; 58]. Formalin-fixed, paraffin-embedded tissue (one representative paraffin block per case containing the complete lesion or the largest part of the lesion resected at surgery) was sectioned at 6 μm and mounted on pre-coated glass slides (Star Frost, Waldemar Knittel GmbH, Barunschweig, Germany). Sections of all specimens were processed for hematoxylin eosin, luxol fast blue and Nissl stainings and neuronal and glial markers for classification and selection.

**Table 1:** Clinical features of cortical development alterations in patients analyzed in this study.

Patient	Age range at surgery (years)	Duration of epilepsy (years)	Diagnosis	Location	Engel’s class
1	10 ≤ 15	8	FCD Ia	Parietal	II
2	15 ≤ 20	10	FCD Ia	Frontal	I
3	>20	16	FCD Ia	Temporal	I
4	15 ≤ 20	11	FCD Ia	Frontal	I
5	>20	17	FCD Ia	Temporal	I
6	15 ≤ 20	12	FCD Ia	Frontal	II
7	0 ≤ 5	4	FCD Ia	Temporal	I
8	0 ≤ 5	3	FCD Ia	Frontal	I
9	0 ≤ 5	1.5	FCD Ia	Multilobar	IV
10	0 ≤ 5	2	FCD Ia	Frontal	I
11	>20	21	FCD IIb	Temporal	I
12	>20	18	FCD IIb	Temporal	I
13	>20	19	FCD IIb	Frontal	I
14	15 ≤ 20	9	FCD IIb	Temporal	I
15	10 ≤ 15	10	FCD IIb	Frontal	I
16	0 ≤ 5	5	FCD IIb	Multilobar	II
17	5 ≤ 10	5	FCD IIb	Multilobar	III
18	5 ≤ 10	7.5	FCD IIb	Frontal	II
19	0 ≤ 5	0.5	FCD IIb	Multilobar	III
20	15 ≤ 20	17	FCD IIb	Multilobar	II
21	0 ≤ 5	0.5	FCD IIb	Multilobar	II
22	>20	21	FCD IIb	Multilobar	III
23	0 ≤ 5	2.5	FCD IIb	Temporal	I
24	10 ≤ 15	15	FCD IIb	Parietal	II
25	10 ≤ 15	6	FCD IIb	Occipital	I
26	5 ≤ 10	5	FCD IIb	Frontal	I
27	0 ≤ 5	0.5	FCD IIa	Occipital	II
28	5 ≤ 10	5	FCD IIa	Temporal	I
29	5 ≤ 10	5	FCD IIa	Multilobar	I
30	0 ≤ 5	3	FCD IIb	Frontal	I

### FCD Neuronal and Organotypic Cultures

Focal cortical dysplasia derived organotypic cultures were obtained as described (Eugène et al., 2014). In brief, resection tissue derived from refractory epilepsy surgery of FCD Type II patients was sliced at 300 μm. Slices were cultured on a transwell semiporous membrane for 7 days and subjected to pharmacological regulation. After incubation, slices were fixed and sectioned for immunofluorescence characterization. Alternatively, proteins were extracted after cell lysis in a buffer containing 50 mM Tris, 0.1% Triton X-100, 1 mM ethylenediaminetetraacetic acid, 1 mM ethylene glycol tetraacetic acid, 50 mM NaF, 10 mM sodium b-glicerophosphate, 5 mM sodium pyrophosphate and 1 mM sodium orthovanadate (pH 7.5) supplemented with a protease inhibitor cocktail (Roche, Basel, Switzerland), 0.1 mM phenylmethane-sulphonylfluoride, 0.1% β-mercaptoethanol and 1 μM microcystin for Western blot analyses.

### CB_1_ Receptor Promoter Transcriptional Assays

Embryonic carcinoma P19 cells were employed in reporter assays as previously described (Díaz-Alonso et al., 2015). Cells were transfected transiently with a construct encoding the -3016 to +142 sequence (referring to the first nucleotide of exon 1) of the human CB_1_ receptor gene promoter fused to the CAT reporter gene (phCB_1_-3016-CAT) (Blázquez et al., 2011). The reporter gene construct was based on the pBLCAT2/pBLCAT3 system, in which the thymidine kinase minimal promoter was replaced for the human CB_1_ receptor promoter upstream of CAT. CAT activity was subsequently analyzed by ELISA following manufacturer’s instructions (Roche, distributed by Sigma #11363727001, Madrid, Spain).

### Immunofluorescence Microscopy

P19 carcinoma cells, organotypic brain slices and FCD samples were fixed and immunofluorescence performed. After blockade with 5% goat serum, overnight incubation at 4°C with the indicated primary antibodies was performed: polyclonal guinea pig anti-CB_1_ receptor (1:500, Frontier Institute, Japan), polyclonal rabbit anti-phosphoS235/S236-S6 ribosomal protein (1:100) and polyclonal rabbit anti-phosphoS240/S244-S6 ribosomal protein (1:800; Cell Signaling Technology, Barcelona, Spain) and monoclonal mouse anti-c-Myc (1:500; Sigma, Madrid, Spain). Specificity of CB_1_ immunoreactivity was confirmed with an additional polyclonal anti-CB_1_ antibody (kindly donated by K. Mackie, Indiana University, Bloomington, IN, United States). The appropriate anti-mouse, rat, guinea pig, and rabbit highly cross-adsorbed AlexaFluor 488, AlexaFluor 546, AlexaFluor 594, and AlexaFluor 647 secondary antibodies (Invitrogen, Carlsbad, CA, United States) were used. Confocal fluorescence images were acquired by using both Leica TCS-SP2 and LAS-X software (Wetzlar, Germany) with a SP2 or a SP8 microscope, respectively, with three passes by Kalman filter and a 1024 × 1024 or a 2048 × 2048 collection box, respectively. Immunofluorescence data were obtained in a blind manner by an independent observer and sample code remained unsealed during the whole data processing and analysis. mTORC1 activation status and CB_1_ immunoreactivity were quantified in a minimum of 500 cells for each FCD patient or control sample. Immunoreactivity was measured using Fiji (ImageJ) software establishing a threshold to measure only specific signal. The resulting binary mask was then used along the built-in measure function to acquire the total integrated gray density among all the pixels inside the binary mask overlayed on top of the original image. The obtained value was then referred to the number of DAPI^+^ cell nuclei present in the optic field. For *in vitro* studies, P19 cells (*n* = 6 independent experiments) and FCD organotypic cultures (*n* = 4 independent FCD cases) were quantified.

### Western Blot Assays

Equal amount of protein samples were electrophoretically separated and transferred to PVDF membranes. After blocking with 5% BSA, membranes were incubated overnight at 4°C with anti-phosphoS235/S236-S6 ribosomal protein (1:1000), anti-phosphoS240/S244-S6 ribosomal protein (1:1000), anti-CB_1_ (1:500) or anti-β-actin (1:5000) primary antibodies. PVDF membranes were then incubated with the corresponding secondary antibodies coupled to horseradish peroxidase. Optical density of the specific immunoreactive band was quantified with Fiji software. The values of pS6 were normalized to those of β-actin in the same membranes.

### DNA Single Nucleotide Polymorphisms Analyses and Sequencing

Genomic DNA was obtained by standard methods and Sequenom SNP analyses were performed by Centro Nacional de Genotipado (CEGEN-PRB2 USC node, Santiago de Compostela, Spain) using the iPlex^®^ Gold chemistry and MassARRAY platform, according to manufacturer’s instructions (Sequenom, San Diego, CA, United States). Genotyping assays were designed using the Sequenom MassARRAY Assay Designer 4.1 software. SNPs were genotyped in three assays, PCR reactions were set up in a 5 μl volume and contained 20 ng of template DNA, 1× PCR buffer, 2 mM MgCl_2_, 500 μM dNTPs and 1 U/reaction of PCR enzyme. A pool of PCR primers (Metabion, Steinkirchen, Germany) was made at a final concentration of each primer of 100 nM. The thermal cycling conditions for the reaction consisted on an initial denaturation step at 94°C for 2 min, followed by 45 cycles of 94°C for 30 s, 56°C for 30 s and 72°C for 1 min, and a final extension step of 72°C for 1 min. PCR products were treated with 0.5 U shrimp alkaline phosphatase, followed by enzyme inactivation to neutralize unincorporated dNTPs.

The iPLEX GOLD reactions were set up in a final 9 μl volume and contained 0.222x iPLEX buffer Plus, 1x iPLEX termination mix and 1x iPLEX enzyme. An extension primer mix (Metabion) was made to give a final concentration of each primer between 0.52 μM and 1.57 μM. The thermal cycling conditions for the reaction included an initial denaturation step at 94°C for 30 s, followed by 40 cycles of 94°C for 5 s, with an internal five cycles loop at 52°C for 5 s and 80°C for 5 s, followed by a final extension step of 72°C for 3 min. The iPLEX Gold reaction products were desalted, dispensed onto a 384 Spectrochip II using an RS1000 Nanodispenser and spectra were acquired using the MA4 mass spectrometer, followed by manual inspection of spectra by trained personnel using MassARRAY Typer software, version 4.0. All assays were performed in 384-well plates, including negative controls and a trio of Coriell samples (Na10830, Na10831, and Na12147) for quality control. Seven samples were tested in duplicate and they were 100% concordant. Genomic DNA sequencing of the CB_1_ receptor gene coding exon was performed by the Sanger method under standard conditions by Secugen (Madrid, Spain).

### Data and Statistical Analyses

Results shown represent the means ± SEM, and the number of experiments is indicated in every case. Statistical analysis was performed with GraphPad Prism 6.07 (GraphPad Software, La Jolla, CA, United States) using one-way ANOVA. A *post hoc* analysis by the Student–Newman–Keuls test was made (Table 2). Disease-Genotype association test was performed by the SNPator online tool (Morcillo-Suarez et al., 2008) to examine genotype and allele frequencies between patients and controls. *P*-values of <0.05 were regarded as statistically significant.

**Table 2:** Statistical analyses.

Figure	Comparison	Statistic value	Significance
Figure 1A	One-way ANOVA	*F* = 22.57	^∗∗∗∗^
	FCDI vs. Co	*q* = 1.930	ns
	FCDII vs. Co	*q* = 7.833	^∗∗∗∗^
	FCDII vs. FCDI	*q* = 7.005	^∗∗∗∗^
Figure 1B	One-way ANOVA	*F* = 24.87	^∗∗∗∗^
	FCDI vs. Co	*q* = 1.013	ns
	FCDII vs. Co	*q* = 8.856	^∗∗∗∗^
	FCDII vs. FCDI	*q* = 7.271	^∗∗∗∗^
Figure 1C	One-way ANOVA	*F* = 24.87	^∗∗∗∗^
	FCDI vs. Co	*q* = 0.7072	ns
	FCDII vs. Co	*q* = 7.399	^∗∗∗∗^
	FCDII vs. FCDI	*q* = 8.187	^∗∗∗∗^
Figure 3A	One-way ANOVA	*F* = 8.749	^∗∗^
	RHEB vs. pCAG-GFP	*q* = 4.450	^∗∗^
	shTSC2 vs. shCo	*q* = 5.700	^∗∗^
Figure 3B	One-way ANOVA	*F* = 2.917	^∗^
	Tbr2 vs. GFP	*q* = 5.083	^∗∗^
Figure 5A	One-way ANOVA	*F* = 16.77	^∗∗∗^
	RAPA vs. VEH	*q* = 7.675	^∗∗∗^
	RAPA vs. HU-210	*q* = 6.139	^∗∗^
Figure 5B	One-way ANOVA	*F* = 7.305	^∗∗∗^
	HU-210 vs. VEH	*q* = 0.2946	ns
	SR1 vs. VEH	*q* = 4.164	^∗^
	RAPA vs. VEH	*q* = 5.153	^∗∗^
	HU-210 + SR1 vs. VEH	*q* = 5.208	^∗∗^

*One-way ANOVA followed by Student–Newman–Keuls *post hoc* test. Statistical significance ^∗∗∗∗^*p* < 0.0001; ^∗∗∗^*p* < 0.001; ^∗∗^*p* < 0.01; ^∗^*p* < 0.05; ns, non-significant.*

## Results

### CB_1_ Cannabinoid Receptor Expression in FCD Is Associated With Overactive mTORC1 Pathway

**FIGURE 1:**
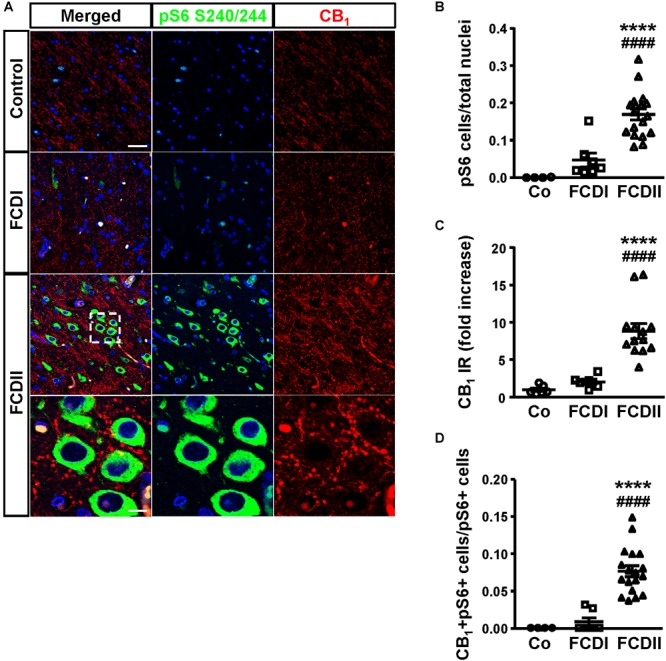
The CB_1_ receptor is enriched in focal cortical dysplasia (FCD) Type II in cells with overactive mTORC1 signaling. **(A)** Representative immunofluorescence images showing the presence of CB_1_ receptors in samples from Type I and II FCD and control brains, revealed with an anti-CB_1_ antibody (red). Cells with over-active mTORC1 signaling are stained with phospho-S6_Ser240/244_ antibody (green). High magnification images of CB_1_ receptor expression associated with mTORC1 overactivation in FCD Type II are shown. **(B,C)** Phospho-S6^+^ cells and CB_1_ receptor immunoreactivity (IR) were quantified in the dysplastic area and referred to total cell number (DAPI counterstaining). Control, FCD Type I and FCD Type II cases (**B**, *n* = 4, 7 and 18, respectively; **C**, *n* = 6, 8, 13). **(D)** CB_1_^+^ phospho-S6^+^ double-labeled cells were quantified and referred to total pS6^+^ cells. Control, FCD Type I and FCD Type II cases (*n* = 4, 7, and 18, respectively). Statistical comparison versus control samples, ^∗∗∗∗^*p* < 0.0001; statistical comparison versus FCD Type I samples, ^####^*p* < 0.0001. Scale bar 45 μm, insets, 10 μm.

**FIGURE 2:**
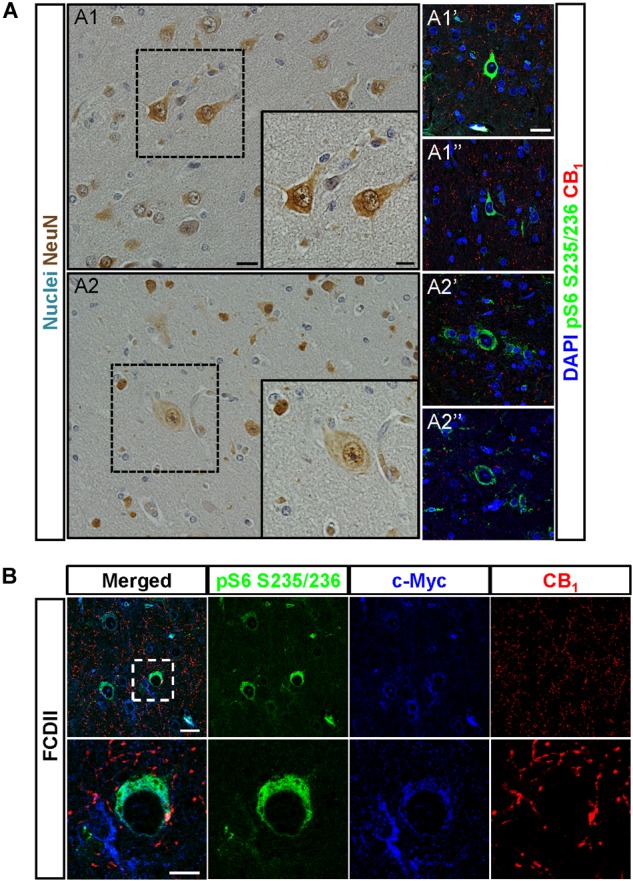
CB_1_ receptors are present in undifferentiated cells. **(A)** Dysplastic neurons **(A1,A1′,A1″**) and balloon cells **(A2,A2′,A2″)** were identified based in morphological criteria in hematoxylin/eosin sections stained with NeuN antibody **(A1,A2)**. CB_1_ receptor expression and mTORC1 activity status were analyzed by immunofluorescence with CB_1_ and phospho-S6 antibodies, respectively **(A1′,A1″,A2′,A2″)**. **(B)** Representative immunofluorescence images showing the presence of CB_1_ receptor (red) in FCD Type II brain cells labeled with the undifferentiated cell marker c-Myc (blue) and active mTORC1 signaling phospho-S6^+^ cells (green). High magnification insets are shown (lower panels). Scale bar: 25 μm, insets, 10 μm.

To assess the pathophysiological relevance of CB_1_ receptor signaling in MCD we analyzed CB_1_ receptor immunoreactivity in control and dysplastic brain areas (Figure 1). FCD cases (*n* = 30) were included with a mean patient age of 11.4 years and a male/female distribution of *n =* 18 and 12, respectively (Table 1). Double immunofluorescence analysis with anti-phospho-S6 (recognizing the phosphoS240/S244 sites) and anti-CB_1_ antibodies confirmed the selective overactivation of the mTORC1 pathway in FCD Type II but not FCD Type I samples (Figures 1A,B). Quantification of CB_1_ immunoreactivity revealed that receptor expression is notably enriched in the dysplastic areas of FCD Type II when compared with control brain tissue, but not in FCD Type I (Figures 1A,C). A more detailed analysis of double immunofluorescence images showed that CB_1_-positive cells largely colocalized with phospho-S6 immunoreactivity in FCD Type II, and CB_1_^+^pS6^+^ cells were highly enriched in the dysplastic areas when compared to control cortical tissue (Figure 1D). Equivalent findings of CB_1_ enrichment in phospho-S6-positive cells were reproduced when using as alternative readout, anti phosphoS235/S236-S6 antibody (Supplementary Figure 1). Moreover, the intensity of phospho-S6 immunoreactivity was selectively increased when comparing FCD Type II and I (1.61 ± 0.14 versus 1.00 ± 0.07, respectively; *p* < 0.05, *n* = 4). Next, we analyzed CB_1_ receptor expression in BCs and dysplastic neurons based in morphological characterization and mTORC1 overactivation (Figure 2A). This indicated that 2.43 ± 0.92 and 0.57 ± 0.29% of total CB_1_ tissue immunoreactivity corresponded to these cell subpopulations, respectively. FCD Type II is characterized by the expression of undifferentiated markers in BC including Sox2, Oct4, Pax6, Tbr1, Otx1, and others (Hadjivassiliou et al., 2010; Orlova et al., 2010; Arai et al., 2012; Yao et al., 2016). Thus, we analyzed the expression of CB_1_ receptors in FCD Type II neurons together with undifferentiated-cell markers. Whereas in FCD Type II sparse c-Myc-positive cells could be detected, this was never the case in the FCD Type I samples. Importantly, c-Myc-positive cells expressed CB_1_ receptors and showed active mTORC1 signaling (pS6^+^) (Figure 2B). Hence, in FCD Type II we determined that 72.74 ± 11.04% c-Myc-positive cells were also phospho-S6 positive.

### CB_1_ Cannabinoid Receptor Expression Is Not Induced by mTORC1 Signaling

**FIGURE 3:**
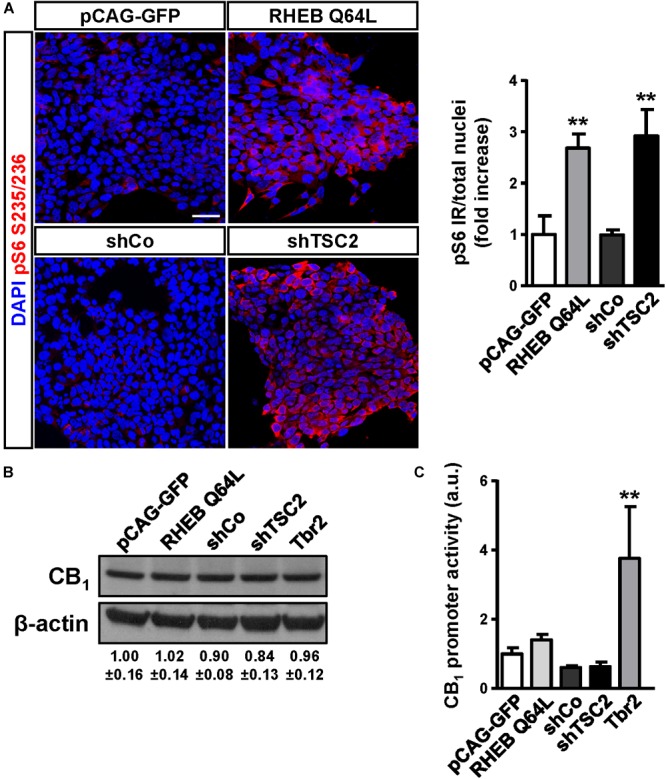
The mTORC1 pathway does not regulate CB_1_ receptor expression. **(A)** Characterization of mTORC1 activation in P19 cells after transfection with the Rheb constitutive active mutant Q64L, shTSC2, a scrambled shRNA encoding vector (shCo), or GFP encoding plasmid. mTORC1 activation was analyzed by means of phospho-S6-positive cells quantification. Scale bar: 50 μm. **(B)** Western blot analysis of CB_1_ receptor levels in the same conditions as above. Representative immunoblot luminograms for CB_1_ and β-actin as loading control are shown and quantification is provided (mean ± SEM, *n* = 3). **(C)** CB_1_ receptor promoter activity was analyzed using a CAT construct 24 h after transfection (*n* = 6). Statistical comparison versus the corresponding control samples, ^∗∗^*p* < 0.01.

To determine if CB_1_ receptor enrichment in FCD lesions was a cause or a consequence of mTORC1 overactivation we analyzed if this signaling pathway can regulate CB_1_ receptor expression. P19 cells were transfected with a plasmid encoding a constitutively active mutant of the mTORC1 upstream activator Rheb (RhebQ64L) or a Tsc2 specific short-hairpin RNA coding plasmid (shTSC2). In these conditions, as compared to control cells, mTORC1 pathway activity increased as evidenced by the strong increase of pS6^+^ immunoreactive cells (Figure 3A). However, under the same conditions of overactive mTORC1 signaling, CB_1_ receptor protein levels were not induced (Figure 3B). We also performed CB_1_ promoter transcriptional assays by co-transfection with a CAT gene reporter in frame with a minimal *CNR1* promoter (Blázquez et al., 2011). Again, in cells with overactive mTORC1 pathway CB_1_ promoter activity was not induced (Figure 3C). As a control of the sensitivity of this assay, P19 cells were transfected with the intermediate progenitor transcription factor Tbr2 (Eomes). The CB_1_ promoter has several putative Tbr2-binding sites [Table 3, Matinspector (Genomatix)], and its expression was indeed sufficient to increase CB_1_ promoter reporter activity (Figure 3C), but failed to increase protein levels (Figure 3B). In summary, these results indicate that the increased CB_1_ receptor levels in FCD are not a direct consequence of an overactive mTORC1 pathway.

**Table 3:** Putative Tbr2 (Eomes) binding sites in the Cnr1 locus identified using MatInspector software.

Species	Gene location	Binding site position		Strand	Matrix similarity
		Start	End		
Human	6q15	88164872	88164900	**+**	0.941
		88166737	88166765	**+**	0.963
		88166645	88166673	**+**	0.989
		88166554	88166568	**+**	0.902
		88168289	88168317	**+**	0.99
Mouse	4A5	33936594	33936608	**–**	0.896
		33925880	33925908	**+**	0.901
		33944561	33944589	**–**	0.999

### Genetic Characterization of the Endocannabinoid System in FCD

The observation that CB_1_ receptor levels are increased in the dysplastic cells of FCD Type II cases prompted us to expand the analyses to other elements of the ECS that may contribute to cannabinoid signaling deregulation. Genomic DNA and messenger RNA from the FCD collection were obtained. Real time PCR expression analysis confirmed increased levels of CB_1_ receptor transcripts in FCD Type II versus control brain extracts (Table 4), further supporting the results obtained at the protein level by immunofluorescence characterization (Figure 1). Transcript levels of other elements of the ECS [DAGL alpha and beta isoforms, MAGL, FAAH and *N*-acyl phosphatidylethanolamine phospholipase D (NAPE-PLD)] were also quantified and no differences were observed (Table 4). To further characterize if cannabinoid signaling alterations may contribute to overactive mTORC1 in FCD Type II, we performed single nucleotide polymorphism (SNP) analysis of various genes of the ECS including the CB_1_ receptor, DAGL alpha and beta, MAGL, FAAH and CB_2_ receptor (Supplementary Table 3). A total 48 SNPs of the ECS were selected, based on previous evidences that point to their potential involvement in different nervous system disorders. Genotype disease association analysis revealed the existence of three polymorphisms in the *DAGLA* gene differentially expressed in FCD Type II versus control specimens (Table 5). The rest of SNPs analyzed for *CNR1, DAGLB, MAGL, FAAH*, and *CNR2* did not show any difference between pathologic samples and controls. To further investigate the potential involvement of CB_1_ receptors in FCD we sequenced the *CNR1* gene exon in the FCD and control genomic DNA samples. *CNR1* exon 1 sequencing revealed normal wild-type sequence in most samples and only rs1049353 SNP (c.1359G>A; p.Thr453) was identified with similar distribution among dysplastic and control DNA.

**Table 4:** Endocannabinoid system elements analyzed by qPCR in FCD Type II and control samples.

Transcript	Condition	Mean	SEM	Significance
CB_1_	Control	1.00	0.2397	^∗^
	FCDII	33.70	5.3080	
DAGLα	Control	1.00	0.0004	ns
	FCDII	0.45	0.1286	
DAGLβ	Control	1.00	0.0106	ns
	FCDII	0.84	0.2274	
MAGL	Control	1.00	0.0005	ns
	FCDII	0.88	0.3061	
NAPE-PLD	Control	1.00	0.0264	ns
	FCDII	0.72	0.1176	
FAAH	Control	1.00	0.0182	ns
	FCDII	0.36	0.1910	

*Statistical comparison *versus* control tissue ^∗^*p* < 0.05. CB_1_ transcript (*n* = 4 and 22, control and FCDII), other endocannabinoid system elements (*n* = 4 and 8 control and FCDII).*

**Table 5:** SNPs analyzed in Focal cortical dysplasia Type II and control brain genomic DNA extracts.

SNP	Alleles	Major allele homozygous (%)		Heterozygous (%)		Minor allele homozygous (%)		Disease association
	Major/Minor	Controls	FCD	Controls	FCD	Controls	FCD	
rs806365	C/T	20.0	36.4	70.0	45.4	10.0	18.2	
rs7766029	T/C	20.0	31.8	50.0	54.5	30.0	13.6	
rs806366	T/C	40.0	31.8	40.0	31.8	20.0	36.4	
rs806368	T/C	60.0	54.5	20.0	36.4	20.0	9.0	
rsl2720071	A/G	60.0	86.4	40.0	13.6	0.0	0.0	
rs4707436	G/A	60.0	59.1	30.0	40.9	10.0	0.0	
rsl049353	G/A	80.0	68.2	10.0	31.8	10.0	0.0	
rs806369	C/T	60.0	45.5	30.0	40.9	10.0	13.6	
rs2023239	T/C	60.0	63.6	40.0	36.4	0.0	0.0	
rsl535255	T/G	70.0	63.6	30.0	36.4	0.0	0.0	
rs806379	A/T	30.0	18.2	60.0	77.3	10.0	4.5	
rs9444584	C/T	60.0	54.5	30.0	45.5	10.0	0.0	
rs9450898	C/T	70.0	63.6	30.0	36.4	0.0	0.0	
rs806380	A/G	60.0	54.5	30.0	40.9	10.0	4.5	
rs6454674	T/G	40.0	63.6	40.0	31.8	20.0	4.5	
rs2180619	A/G	30.0	31.8	50.0	45.5	20.0	22.7	
rs4963304	G/A	10.0	68.2	50.0	31.8	40.0	0.0	^∗^
rs7931563	T/G	50.0	40.9	50.0	40.9	0.0	18.2	
rs7942387	C/A	90.0	100.0	10.0	0.0	0.0	0.0	
rsl98430	C/T	30.0	77.3	40.0	22.7	30.0	0.0	^∗^
rsl98444	T/C	40.0	4.5	60.0	45.5	0.0	50.0	^∗^
rs34365114	G/A	100.0	95.5	0.0	4.5	0.0	0.0	
rsl44674730	C/T	100.0	100.0	0.0	0.0	0.0	0.0	
rsl43650244	AAA/-	90.0	100.0	10.0	0.0	0.0	0.0	
rsl87296513	C/T	100.0	100.0	0.0	0.0	0.0	0.0	
rs3813518	G/A	50.0	77.3	50.0	18.2	0.0	4.5	
rs3813517	A/G	100.0	95.5	0.0	4.5	0.0	0.0	
rs836559	C/G	10.0	36.4	40.0	45.4	50.0	18.2	
rs2303361	T/C	40.0	72.8	30.0	22.7	30.0	4.5	
rs76802560	G/T	100.0	100.0	0.0	0.0	0.0	0.0	
rs6801421	G/A	90.0	63.6	10.0	36.4	0.0	0.0	
rs72969613	C/T	100.0	100.0	0.0	0.0	0.0	0.0	
rs4881	A/G	70.0	81.8	20.0	18.2	10.0	0.0	
rsll5970931	A/G	100.0	100.0	0.0	0.0	0.0	0.0	
rs932816	G/A	80.0	50.0	20.0	45.5	0.0	4.5	
rs4141964	G/A	30.0	40.9	30.0	40.9	40.0	18.2	
rs324420	C/A	30.0	59.1	50.0	22.7	20.0	18.2	
rs324419	G/A	80.0	81.8	20.0	18.2	0.0	0.0	
rs2295632	C/A	30.0	54.5	40.0	27.3	30.0	18.2	
rsl2029329	G/C	30.0	54.5	40.0	27.3	30.0	18.2	
rsl2744386	C/T	10.0	31.8	70.0	50.0	20.0	18.2	
rsll30321	A/G	20.0	27.3	80.0	54.5	20.0	18.2	
rsll06	G/C	20.0	27.3	80.0	54.5	20.0	18.2	
rs2229579	C/T	70.0	63.6	30.0	27.3	0.0	9.1	
rs2501431	A/G	20.0	27.3	80.0	54.5	20.0	18.2	
rs41311993	G/T	100.0	100.0	0.0	0.0	0.0	0.0	
rs35761398	CC/TT	20.0	23.8	80.0	57.2	20.0	19.0	
rs2501432	C/T	20.0	27.3	80.0	54.5	20.0	18.2	

*Major and minor allele’s distribution in control and focal cortical dysplasia (FCD) Type II. Disease association ^∗^*p* < 0.05.*

In summary, these results suggest that the DAGLα-evoked generation of the endocannabinoid 2-AG might be altered in FCD Type II and can contribute to its etiopathology. However, the exact contribution of endocannabinoid tone alterations in MCD would require more complex genetic studies to identify its potential association with the origin of the disease.

### CB_1_ Cannabinoid Receptor Crosstalk With the mTORC1 Pathway in FCD Resections

**FIGURE 4:**
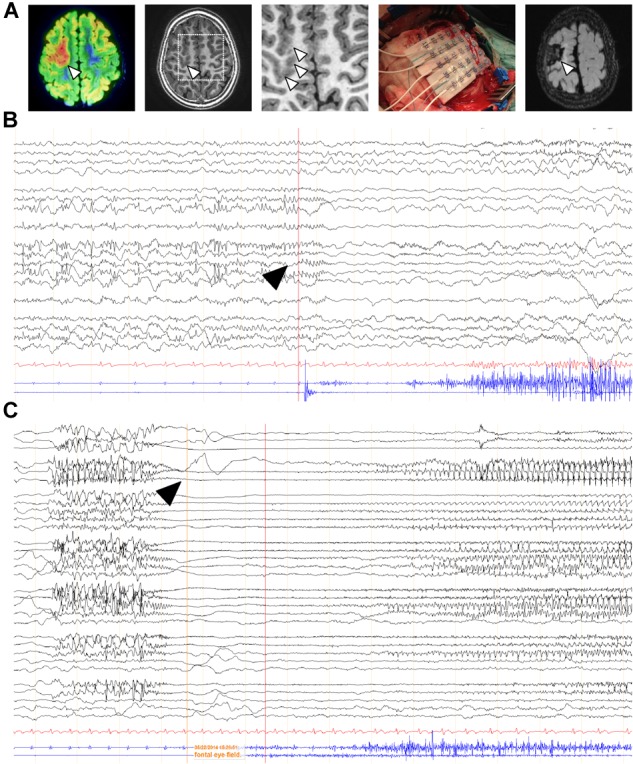
EEG characterization of a representative FCD case subject to surgery. **(A)** FDG-PET scan co-registered with 3T MRI of a 5-year-old child affected by a “malignant rolandic epilepsy” with daily motor seizures and *Epilepsia Partialis Continua.* Hypermetabolic FDG-PET focus (arrowheads). Small focal cortical dysplasia involving the left rolandic region and superior frontal gyrus (arrowhead) and MRI 1 year after epilepsy surgery. **(B,C)** Scalp and intracraneal EEG recording, respectively, of the previous patient, using a combination of subdural electrodes and depth electrodes, prior resection of the epileptogenic zone. Arrowheads, indicate epileptiform activity.

Additional specimens derived from surgical resection for intractable epilepsy were analyzed *ex vivo* for mechanistic studies. 3T magnetic resonance imaging (MRI), fluorodeoxyglucose (FDG)-PET scan, scalp electroencephalography (EEG) recording and invasive neurophysiological studies were employed to identify the origin of epileptic seizures (Figure 4A). Representative images of one case prior and after surgery are shown. In this particular patient, a 5-year-old male, a small FCD Type II involving the left rolandic region and superior frontal gyrus (arrowheads, Figure 4A) was associated with daily focal motor seizures and *Epilepsia Partialis Continua* involving the left arm and the face. Scalp EEG analyses revealed continuous focal epileptiform discharges (arrowhead, Figure 4B) in accordance with a hypermetabolic FDG-PET focus. Intracraneal EEG exploration of the dysplastic lesion, using a combination of subdural electrodes and depth electrodes, better defined a characteristic EEG pattern, indicative of FCD. This characteristic EEG pattern shows continuous repetitive burst of epileptiform activity turning into focal EEG ictal patterns (arrowhead, Figure 4C), in association with the onset of clinical seizure signs. After tailored resection of the epileptogenic zone, 1 year follow-up after surgery revealed a seizure-free clinical status, MRI and EEG showed absence of the lesion and normalization of hyperexcitability.

**FIGURE 5:**
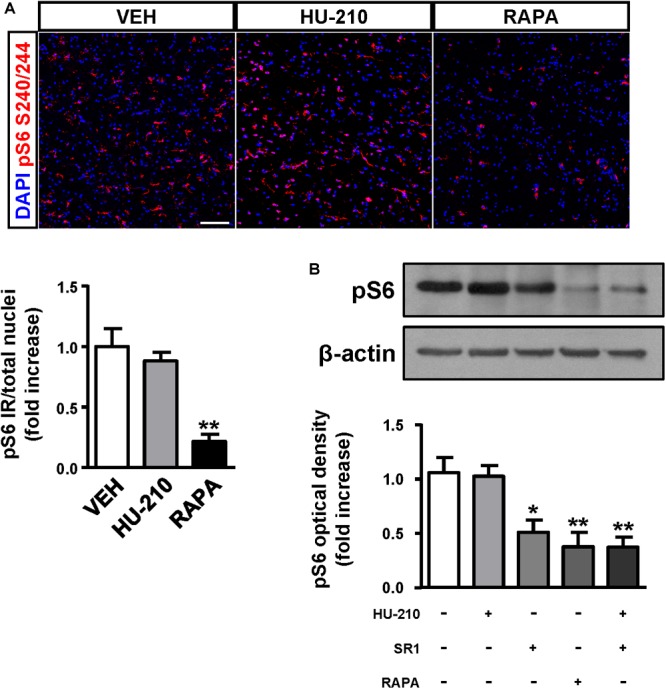
CB_1_ receptors blockade attenuates mTORC1 overactivation in FCD organotypic cultures. Organotypic cultures of FCD resections were cultured 7 days *in vitro* and exposed to the CB_1_ receptor agonist HU-210 (1 μM), and rapamycin (1 μM) 90 min. **(A)** Representative images of immunofluorescence characterization with phospho-S6_S240/244_ antibody were quantified and phosphoS6 immunoreactivity was referred to the total number of cells revealed by DAPI counterstaining. **(B)** Western blot analysis of phospho-S6_S240/244_ levels was performed in slice extracts after 90 min incubation with HU-210 alone or together with SR141716 (25 μM), SR141716 or rapamycin (*n* = 4 experiments). Statistical comparison *versus* vehicle samples, ^∗^*p* < 0.05; ^∗∗^*p* < 0.01. Scale bar: 80 μm.

Considering the regulatory role of CB_1_ receptors in cortical progenitor cell identity via mTORC1 signaling (Díaz-Alonso et al., 2015), we next sought to investigate the impact of the receptor in FCD-derived neurons. We obtained FCD Type II organotypic cultures derived from fresh resections that were maintained for 7 days *in vitro* and subjected to pharmacological manipulation. Quantification of phospho-S6 immunoreactivity revealed that CB_1_ receptor activation with the cannabinoid agonist HU-210 was without effect on mTORC1 activation, whereas the mTORC1 inhibitor rapamycin was effective in reducing mTORC1 overactivation (Figure 5A). Western blot analysis confirmed that CB_1_ receptor agonism did not influence mTORC1 activation, whereas the CB_1_ inverse agonist SR141716 (rimonabant), as well as rapamycin, reduced mTORC1 activation as assessed by phosphoS240/244-S6 levels (Figure 5B). Equivalent results were obtained with the alternative phosphoS235/236-S6 antibody (Supplementary Figure 2). Overall, these results indicate that inhibition, but not activation of the CB_1_ receptor, may tune the overactive mTORC1 pathway found in FCD Type II dysplastic brain.

## Discussion

In the present study, we characterized the expression and function of the ECS to assess its potential contribution to the etiopathology of MCDs, in particular FCD Type II. Our results reveal a striking increase in CB_1_ receptor expression levels in FCD Type II, and this enrichment occurs in neurons with overactive mTORC1 signaling. In addition, characterization of genomic DNA in the dysplastic brain resections showed an enrichment of three SNPs in the *DAGLA* gene in FCD Type II versus controls. The SNPs analyzed in other ECS element genes did not show any difference between groups. In addition, sequencing of the *CNR1* coding exon did not reveal any SNP or mutation differentially present in FCD Type II versus control brains.

Presynaptic CB_1_ receptors engaged by retrograde endocannabinoid messengers constitute an efficient regulatory mechanism of excessive neurotransmitter release. Hence, endocannabinoid signaling is a crucial pathway controlling neuronal activity and its activation or blockade modulates seizures and epilepsy development (Soltesz et al., 2015). Patients of temporal lobe epilepsy have decreased CB_1_ receptor expression (Ludányi et al., 2008; Goffin et al., 2011). Likewise, selective loss of function of presynaptic CB_1_ receptors in projection neuron populations of the mouse brain results in an imbalance of the excitatory/inhibitory tone and a higher susceptibility to seizures (Monory et al., 2006). Thus, the enrichment in CB_1_ receptor expression in FCD lesions could represent a compensatory mechanism to attenuate the imbalance of excitatory/inhibitory neuronal activity. In addition, gene expression assays indicate that, at least for the hCB_1_ receptor promoter employed (-3016 to +142 bp sequence), is not directly induced by overactive mTORC1 pathway, indicating that other signaling events control CB_1_ receptor expression in this context.

SNPs analyses and genomic DNA sequencing of the *CNR1* exon did not reveal any mutation associated with FCD Type II. Alternatively, the potential involvement of 2-AG metabolism in FCD Type II is suggested by the existence of a selective enrichment in three SNPs of the *DAGLA* gene. The impact of the *DAGLA* SNPs found in our study in 2AG production or DAGL regulation, is yet unknown as they correspond to non-coding regions. Nevertheless *DAGLA* transcript levels were slightly reduced in FCD when compared to control tissue. These results are in agreement with a recent study that found *DAGLA* polymorphisms associated with neurodevelopmental disorders and seizures (Smith et al., 2017), while CB_1_ receptor associated with pain sensitivity, sleep, memory or anxiety, but not seizures. CB_1_ receptors are coupled to the mTORC1 signaling pathway at early stages of neocortex formation (Díaz-Alonso et al., 2015) as well as in the adult brain (Puighermanal et al., 2009). We therefore analyzed CB_1_ receptor downstream signaling in FCD-derived organotypic slices. The CB_1_ agonist HU-210 was unable to further increase mTORC1 activation, reflecting that the pathway is already overactive. Remarkably, the CB_1_ inverse agonist rimonabant was efficient in reducing phospho-S6 levels. This is of interest, as dampening overactive CB_1_ receptor activity with rimonabant in other settings as the Fmr1 knockout mice efficiently decreases exacerbated mTORC1 activity and symptoms (Busquets-Garcia et al., 2013). Thus, these results point to a basal cannabinoid signaling tone that sustains exacerbated mTORC1 activity in FCD Type II. The ribosomal S6 protein is regulated by phosphorylation at multiple sites (Meyuhas, 2015). S6 phosphorylation at Ser240/244 is selectively mediated by S6K1/2 providing a better readout of mTORC1 upstream activation, while Ser235/236 phosphorylation is regulated by different signaling pathways (cAMP/PKA, casein kinase 1, MAPK-activated protein kinase-1 and mTORC1/S6K1/2). Initial studies revealed increased levels of phosphoS235/236-S6 protein in FCD and TSC (Baybis et al., 2004; Aronica et al., 2007). More recently, somatic mutations of MTOR signaling pathway and other upstream regulators PI3K/Akt, TSC1, TSC2, DEPDC5 have been demonstrated in FCD Type II (Jansen et al., 2015; Lim et al., 2015; Ricos et al., 2016). Hence, phosphorylation of S6 protein by different mechanisms may have different functional consequences in FCD and may differ among neural cell types (Ljungberg et al., 2006; Biever et al., 2015). Considering the finding that CB_1_ receptor antagonism attenuates the phosphorylation of the ribosomal S6 protein at different amino acids (Ser235/236 and Ser240/244) it can be predicted that different CB_1_ downstream signaling effectors: cyclic AMP-mediated and cAMP-independent (PI3K/Akt-mediated) contribute to its regulation. Hence, regulation of cannabinoid signaling constitutes and attractive target for various mTOR-associated disorders and symptoms -the so-called “mTORopathies”. Cannabidiol, a non-psychotomimetic cannabinoid with multiple targets, marketed as Epidiolex (GW Pharma), has demonstrated its efficacy as antiepileptic drug in refractory epilepsy including TSC, Dravet and Lennox-Gastaut syndromes, and is under clinical trial for FCDs (Devinsky et al., 2015; Hess et al., 2016; Thiele et al., 2018).

Interestingly, FCD type II is believed to be originated from the dorsal telencephalic progenitor cell compartment and their excitatory neuronal progeny (D’Gama et al., 2017). Noteworthy, in mTORopathies experimental models of MCDs unbalanced deep and upper layer neuronal development occurs as consequence of aberrant expression of neural fate determinants. Hence, a contribution of CB_1_ receptor signaling to neural precursor alterations responsible for FCD Type II cannot be excluded. The consequences of CB_1_ receptor activity during development and later in the adult brain can be intrinsically different. During embryonic development, CB_1_ receptor controls radial glial to intermediate progenitor cell transition via mTORC1 signaling (Díaz-Alonso et al., 2015), and later, at postmitotic stages, tunes deep cortical neuronal differentiation (Mulder et al., 2008; Díaz-Alonso et al., 2012). Thus, CB_1_ receptor activity during embryonic stages controls progenitor proliferation and neuronal differentiation, and alterations in cannabinoid signaling have the potential to evoke long-term neuronal plasticity and abnormalities underlying FCD neuronal hyperexcitability. In animal models, conditional ablation of the CB_1_ receptor during embryonic development results in increased RhoA levels, ectopic neuron accumulations and increased seizure susceptibility (Díaz-Alonso et al., 2017). Noteworthy, RhoA knockdown prevents brain hyperexcitability and projection neuron alterations induced by CB_1_ receptor ablation. Therefore, either hyper- or hypoactive endocannabinoid signaling during cortical development can be responsible for neuronal differentiation and positioning deficits contributing to MCDs. Later, in adult brain, when neuronal activity is established, the neuromodulatory function of the ECS takes place. The precise consequences of CB_1_ receptor activity in the hyperexcitability neuronal circuit of the dysplastic brain remain unknown. Among other mechanisms involved in FCD, decreased hyperpolarization-activated non-specific cation currents contribute to pyramidal layer V hyperexcitability (Albertson et al., 2011). Interestingly, a particular pool of somatodendritic CB_1_ receptors can regulate Ih currents and this explains in turn some of the cognitive consequences of CB_1_ signaling (Maroso et al., 2016).

This study highlights the pathological implications of altered developmental cannabinoid signaling in refractory epilepsy. Characterization of epileptogenic FCD tissue from palliative surgery and dysplastic-derived organotypic cultures indicates that increased CB_1_ receptor signaling may constitute a compensatory mechanism to counteract FCD Type II hyperexcitability, and its antagonism can dampen mTORC1 overactivation. We anticipate that in the near future new genetic linkage association analyses using larger cohorts of patients with pediatric epilepsy could provide further support to cannabinoid signaling deregulation as a causal mechanism underlying refractory epilepsy.

## Ethics Statement

This study was carried out in accordance with the recommendations of “Hospital Universitario Niño Jesús Madrid, Ethic committee” with written informed consent from all subjects. All subjects gave written informed consent in accordance with the Declaration of Helsinki. The protocol was approved by the “Ethic committee of Hospital Universitario Niño Jesús Madrid”.

## Author Contributions

DG-R, JD-A, ZO, AdS-Q, JP-L, JA, and CJ obtained the samples, processed them, and performed the experiments. IdP, VM-C, EA, and MP-J were in charge of resection handling and determination of the dysplastic areas that were analyzed. MG, MP-J, and IG-R designed the study, analyzed the data, and wrote the manuscript.

## Conflict of Interest Statement

The authors declare that the research was conducted in the absence of any commercial or financial relationships that could be construed as a potential conflict of interest.

## Abbreviations

CAT: chloramphenicol acetyltransferase
DAGL: diacylglycerol lipase
ECS: endocannabinoid system
FAAH: fatty acid amide hydrolase
FCD: focal cortical dysplasia
MAGL: monoacylglycerol lipase
MCD: malformation of cortical development
mTORC1: mammalian target of rapamycin complex 1
TSC: tuberous sclerosis complex
