# Supplementary figures and images for: Contribution of Altered Endocannabinoid System to Overactive mTORC1 Signaling in Focal Cortical Dysplasia
Source: Front Pharmacol. 2019 Jan 9;9:1508. doi: 10.3389/fphar.2018.01508 (PMC6334222; doi:10.3389/fphar.2018.01508)

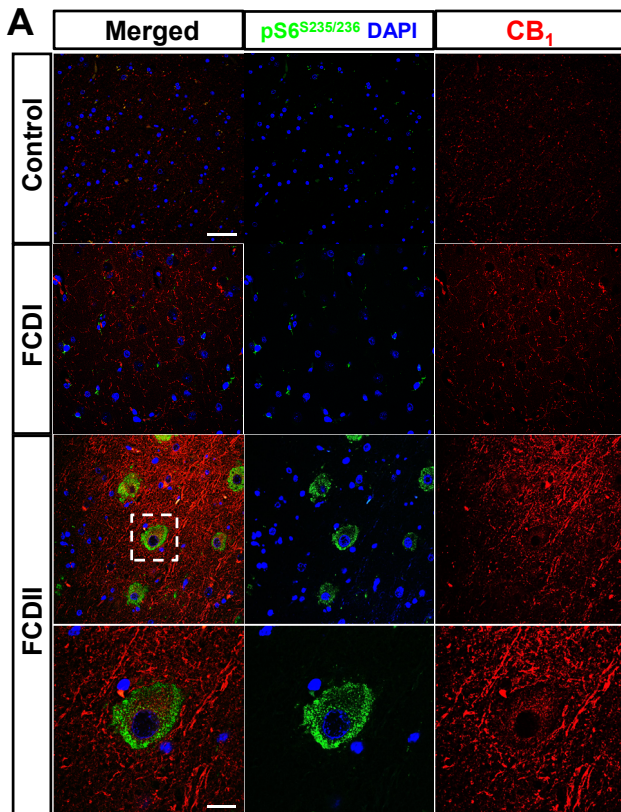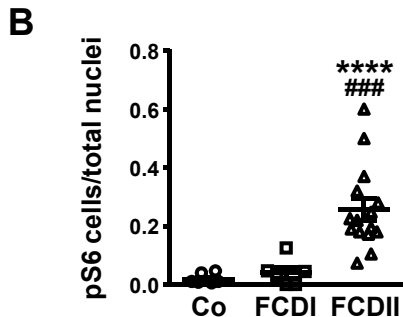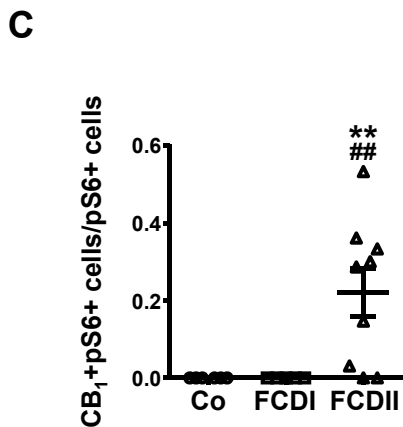

Supplementary Figure 1

Supplement: Supplementary file 1 [file Image_1.pdf]

**A**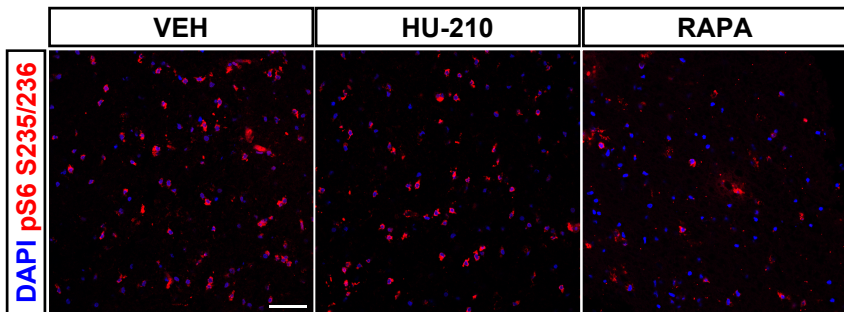**B**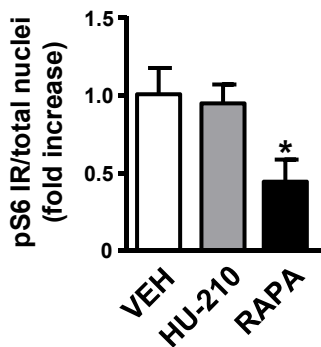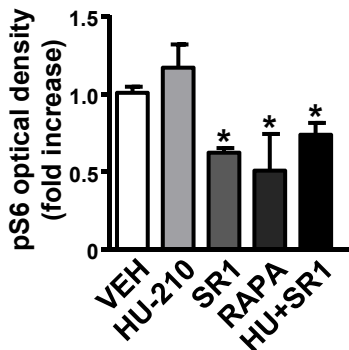

**Supplementary Figure 2**

Supplement: Supplementary file 2 [file Image_2.pdf]
