# Supplementary material for: Contribution of Altered Endocannabinoid System to Overactive mTORC1 Signaling in Focal Cortical Dysplasia
Source: Front Pharmacol. 2019 Jan 9;9:1508. doi: 10.3389/fphar.2018.01508 (PMC6334222; doi:10.3389/fphar.2018.01508)
Supplement: Supplementary file 3 [file Data_Sheet_1.pdf]

Supplementary Figure 1. The CB_1 receptor is enriched in focal cortical dysplasia type II in cells with overactive mTORC1 signalling. *(A)* Representative immunofluorescence images showing the presence of CB_1 receptors in samples from Type I and II focal cortical dysplasia (FCD) and control brains, revealed with an anti-CB_1 antibody (red). Cells with over-active mTORC1 signalling are stained with phospho-$S6_{Ser235/236}$ (green). High magnification images of CB_1 receptor expression associated with mTORC1 overactivation in FCD Type II are shown. *(B)* phospho-$S6^+$ cells were quantified in the dysplastic area and referred to total cell number (DAPI counterstaining). Control, FCD Type I and FCD Type II cases. *(C)* CB_1^+ phospho-$S6^+$ double-labelled cells were quantified and referred to total $pS6^+$ cells. Control, FCD Type I and FCD Type II cases (n= 6, 8 and 9, respectively). Statistical comparison versus control samples, ****, p<0.0001; **, p<0.01; statistical comparison versus FCD Type I samples, ###, p<0.001; ##, p<0.01. Scale bar 25 μm, insets, 10 μm.

Supplementary Figure 2. CB_1 receptors blockade attenuates mTORC1 overactivation in FCD organotypic cultures. Organotypic cultures of FCD resections were cultured 7 days in vitro and exposed to the CB_1 receptor agonist HU-210 (1 μM), and rapamycin (1 μM) 90 min. (*A*) Representative images of immunofluorescence characterization with phospho-$S6_{Ser235/236}$ antibody. Phospho-$S6^+$ cells were quantified and referred to the total number of cells revealed by DAPI counterstaining. (*B*) Western blot analysis of phospho-$S6_{Ser235/236}$ was performed in slice extracts after 90 min incubation with HU-210 alone or together with SR141716 (25 μM), SR141716 or rapamycin (n= 4 experiments). Statistical comparison *versus* control samples, *, p <0.05. Scale bar: 50 μm.

Supplementary Table 1. SNPs analysed in Focal cortical dysplasia Type II and control brain genomic DNA extracts.

Gene	SNP	Functional Consequence
CNR1	rs806365	unknown
CNR1	rs7766029	unknown
CNR1	rs806366	unknown
CNR1	rs806368	utr variant 3 prime
CNR1	rs12720071	utr variant 3 prime
CNR1	rs4707436	utr variant 3 prime
CNR1	rs1049353	synonymous codon
CNR1	rs806369	intron variant, upstream variant 2KB
CNR1	rs2023239	intron variant
CNR1	rs1535255	intron variant
CNR1	rs806379	intron variant
CNR1	rs9444584	intron variant
CNR1	rs9450898	intron variant
CNR1	rs806380	intron variant
CNR1	rs6454674	intron variant, utr variant 5 prime
CNR1	rs2180619	upstream variant 2KB
DAGLA	rs4963304	upstream variant 2KB
DAGLA	rs7931563	upstream variant 2KB
DAGLA	rs7942387	upstream variant 2KB
DAGLA	rs198430	synonymous codon
DAGLA	rs198444	synonymous codon
DAGLA	rs34365114	synonymous codon
DAGLA	rs144674730	missense
DAGLB	rs143650244	downstream variant 500B
DAGLB	rs187296513	downstream variant 500B
DAGLB	rs3813518	downstream variant 500B
DAGLB	rs3813517	downstream variant 500B
DAGLB	rs836559	utr variant 3 prime
DAGLB	rs2303361	missense
MGLL	rs76802560	downstream variant 500B
MGLL	rs6801421	downstream variant 500B
MGLL	rs72969613	utr variant 3 prime
MGLL	rs4881	synonymous codon
MGLL	rs115970931	utr variant 3 prime
FAAH	rs932816	upstream variant 2KB
FAAH	rs4141964	intron variant
FAAH	rs324420	missense
FAAH	rs324419	synonymous codon
FAAH	rs2295632	downstream variant 500B
FAAH	rs12029329	downstream variant 500B
CNR2	rs12744386	unknown
CNR2	rs1130321	utr variant 3 prime
CNR2	rs1106	utr variant 3 prime
CNR2	rs2229579	missense
CNR2	rs2501431	synonymous codon
CNR2	rs41311993	missense
CNR2	rs35761398	missense
CNR2	rs2501432	missense
